# Autolysis of *Pichia pastoris* induced by cold

**DOI:** 10.1186/s13568-017-0397-y

**Published:** 2017-05-12

**Authors:** Yaneth Bartolo-Aguilar, Luc Dendooven, Cipriano Chávez-Cabrera, Luis B. Flores-Cotera, María E. Hidalgo-Lara, Lourdes Villa-Tanaca, Rodolfo Marsch

**Affiliations:** 1 Department of Biotechnology and Bioengineering, Cinvestav-IPN, Av. Instituto Politécnico Nacional 2508, Col. San Pedro Zacatenco, 07360 Gustavo A. Madero, CDMX Mexico; 2 Department of Microbiology, Escuela Nacional de Ciencias Biológicas del IPN, Prol. Carpio y Plan de Ayala S/N Col. Santo Tomás, 11340 Miguel Hidalgo, CDMX Mexico

**Keywords:** Autolysis, Cold-shock promoter, Glucanase, *Saccharomyces cerevisiae*, *Pichia pastoris*, White biotechnology

## Abstract

The production of recombinant biopharmaceutical proteins is a multi-billion dollar market. Protein recovery represents a major part of the production costs. *Pichia pastoris* is one of the microbial systems most used for the production of heterologous proteins. The use of a cold-induced promoter to express lytic enzymes in the yeast after the growth stage could reduce protein recovery costs. This study shows that a cold-shock can be applied to induce lysis of the yeast cells. A strain of *P. pastoris* was constructed in which the endogenous *eng* gene encoding a putative endo-β-1,3-glucanase was overexpressed using the cold-shock induced promoter of the *cctα* gene from *Saccharomyces cerevisiae*. In the transgenic *P. pastoris*, the expression of *eng* increased 3.6-fold after chilling the cells from 30 to 4 °C (cold-shock stage) followed by incubation for 6 h (*eng* expression stage). The culture was heated to 30 °C for 6 h (ENG synthesis stage) and kept at 37 °C for 24 h (lysis stage). After this procedure the cell morphology changed, spheroplasts were obtained and cellular lysis was observed. Thus, a clone of *P. pastoris* was obtained, which undergoes autolysis after a cold-shock.

**Electronic supplementary material:**

The online version of this article (doi:10.1186/s13568-017-0397-y) contains supplementary material, which is available to authorized users.

## Introduction

The development of recombinant proteins has become one of the fastest growing areas in biotechnological and pharmaceutical industries (Rader 2013; Tang and Zhao 2009; Wagner and Alper 2016). Every year, the Food and Drug Administration of the United States approves new biopharmaceutical products that cover a wide range of innovative products for health care. For instance, of the 18 products approved in 2012, eight were recombinant proteins, suggesting that the production of recombinant proteins with pharmaceutical use is an increasing commercial activity (Rader 2013). The methylotrophic yeast *Pichia pastoris* has been used widely as an expression system for recombinant protein production offering fast growth rates in low cost media, relatively fast expression times, high protein yields, ease of genetic manipulation and a post-translational processing system similar to that of Mammalia (Ahmad et al. 2014; Athmaram et al. 2013; Byrne 2015). The production of recombinant proteins includes commonly processing steps such as development and culture of the strain, extraction, purification and formulation of the recombinant protein (Looser et al. 2015; Wolf and Reichl 2011). The extraction and purification of a recombinant protein may contribute to 80% or more of the total production cost (Ahuja 2000). Accordingly, reducing the cost of the extraction and purification is of great economic importance. Cells have to be lysed often by drastic methods to recover intracellular proteins and this can affect the product and thus the yield (Garcia-Ortega et al. 2015). A major impediment to reduce production costs of recombinant proteins is the availability of a low-cost recovery technology that does not affect adversely the protein and/or the environment (Balasundaram et al. 2009). A low cost alternative with little or no environmental impact would be to use autolytic strains. In the wine making industry natural or genetically obtained autolytic *Saccharomyces* strains are used to improve wine quality and facilitate certain parts of the wine making process (Grossmann et al. 2011). Autolysis of *Saccharomyces* cells in this process depends on autophagy and degradation of cell constituents, including proteins (Alexandre and Guilloux-Benatier 2006; Cebollero et al. 2005; Cebollero and Gonzalez 2006; Grossmann et al. 2011). Since recombinant protein processes are intended to produce proteins, the above-mentioned strains cannot be used. A possible solution might be the use of enzymes that degrade yeast cell wall constituents, so that the cell wall becomes fragile, facilitating cell lysis, but avoiding protein degradation.

The inner layer of the yeast cell wall provides rigidity to the cell and consists of a glucan-net with small spots of chitin (Levin 2011). The glucan-net is formed by long chains of β-1,3-glucan with chain branches attached through β-1,6 linkages (Orlean 2012). Endo-β-1,3-glucanases can degrade the glucan-net of *S. cerevisiae* cell wall (Baladrón et al. 2002; Ferrer et al. 1996; Tanaka and Phaff 1965) and they have been used to make the yeast cell wall fragile in certain production processes (Cheng et al. 2013). Moreover, *eng* from *P. pastoris* encodes for a secreted putative endo-β-1,3-glucanase (ENG; accession no. NC_012963) that may be suitable to make the cell wall fragile. In this work, the promoter P*cct*α from *S. cerevisiae*, i.e. a promoter induced by a cold-shock (Al-Fageeh and Smales 2006; Somer et al. 2002), was selected to control *eng* expression from *P. pastoris*. This promoter controls the expression of the alpha subunit of chaperonin-containing T-complex (CCTα) and it was shown previously that a cold-shock increases 3–4 times the expression of CCTα (Somer et al. 2002).

The aim of this work was to show that a transgenic strain of *P. pastoris* could undergo autolysis after induction by a cold-shock. The endogenous *eng* gene of *P. pastoris* was overexpressed under the control of the P*cct*α promoter from *S. cerevisiae*. This promoter was induced by cold-shock, and it was shown that this weakens the yeast cell wall, promotes the formation of spheroplasts and induces cell lysis. This approach can be useful to facilitate the purification of recombinant proteins in industrial processes.

## Materials and methods

### Biological materials

References for the nucleotide sequences used in this work are given in Table 1. They were all amplified by PCR. The cold induced promoter of the alpha subunit of chaperonin-containing T-complex (P*cct*α, chromosome IV, accession number NC_001136, nt: 886842-887284) was amplified from *S. cerevisiae* S288C. The strain was obtained from the National Collection of Microbial Cultures (Cinvestav-IPN, Mexico). *Pichia pastoris* GS115 was purchased from Invitrogen (Carlsbad, CA, USA). This strain was used for the final strain construction as well as the source of the gene encoding for a putative endo-β-1,3-glucanase (chromosome 1, accession number NC_012963; GeneID: 8197918). The functional gene *leu2* encoding a β-isopropylmalate dehydrogenase (β-IMDH) that catalyzes the third step in the leucine biosynthetic pathway (chromosome 3, accession number NC_012965; GeneID: 8199325) was also obtained from *P. pastoris* GS115. *Pichia pastoris* CL2 (deposition number CDBB-L-1984) was constructed in this work and deposited at the “*Colección Nacional de Cepas Microbianas y Cultivos Celulares*” Cinvestav-IPN (Mexico). The plasmid pGAPZαA (Invitrogen) was used as the source to amplify the region *aox1* TT of the transcription terminator of *aox1,* a fragment containing the *tef1* promoter (P_*tef1*_) from *S. cerevisiae*, the synthetic bacterial promoter of EM7 (P_EM7_), the bleomycin resistance gene (*ble*) from *Streptoalloteichus hindustanus* and the transcription terminator of *cyc1* (*cyc1* TT) from *S. cerevisiae*. *Escherichia coli* TOP10 was purchased from Invitrogen and used as plasmid host.

**Table 1:** Sequences used in this study

Sequence	Description	Source	Amplicon	Reference
P*cct*α	Alpha subunit promoter from cytoplasmic chaperonin CCT	*S. cerevisiae*	YL1	Chromosome IV, Accession No. NC_001136, nt: 886842-887284
*eng*	Gene encoding a putative endo-β-1,3-glucanase	*P. pastoris*	YL2	Chromosome 1, Accession No. NC_012963; GeneID: 8197918
*aox1* TT	*aox1* transcription termination region	*P. pastoris*	YL3	Online catalog. pGAPZα A, B, and C. *Pichia* expression vectors for constitutive expression and purification of recombinant proteins (Invitrogen, CA, USA) https://www.thermofisher.com/order/catalog/product/V20020
P_t*ef1*_	Eukaryotic constitutive promoter	*S. cerevisiae*	YL4	
P_EM7_	Prokaryotic constitutive promoter	Synthetic		
*ble*	Gene encoding a protein that confers resistance to zeocin	*S. hindustanus*		
*cyc1* TT	End 3′ of the gene *cyc1* for transcription termination	*S. cerevisiae*		
*leu2*	Functional gene of the β-isopropylmalate dehydrogenase (IMDH)	*P. pastoris*	YL5	Chromosome 3, Accession No. NC_012965; GeneID: 8199325

### Propagation of the strains and DNA isolation

*Saccharomyces cerevisiae* was grown in YM medium (3 g malt extract, 5 g peptone, and 10 g glucose per liter), while *P. pastoris* was cultived in YPD medium (10 g yeast extract, 20 g peptone, 20 g dextrose per liter). The YM and YPD were supplemented with 15 g agar for the solid media. Genomic DNA from both *P. pastoris* and *S. cerevisiae* was extracted from cell cultures in the exponential growth phase, and purified using the Qiagen Dneasy™ Blood & Tissue Kit (Valencia, CA, USA) according to the instructions of the manufacturer.

*Escherichia coli* was grown in LB medium (Sambrook and Russell 2001). Plasmid DNA was purified using a modification of the method of Birnboim and Doly (Sambrook and Russell 2001).

### Construction of plasmid pLGC09

**Fig. 1:**
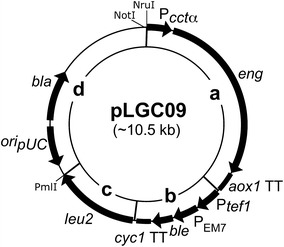
Structure of pLGC09. Plasmid pLGC09 comprises three modules: **a** the lytic regulated module is composed of the promoter of chaperonin CCT one complex (P*cct*α) that drives expression of *eng* in *P. pastoris* and it is finished by *aox1* transcription termination (*aox1* TT) region; **b** the selection marker module is composed of the translational elongation factor 1 gene promoter (P_*tef1*_) and of the EM7 synthetic prokaryotic promoter (P_EM7_) that drive expression of the *ble* gene in *P. pastoris* and *E. coli* respectively, these expressions are finished by *cyc1* transcription termination region (*cyc1* TT); **c** the homologous recombination module is composed of the *leu2* functional gene including its promoter and transcription termination region; and **d** the replicon pCR^®^4Blunt-TOPO^®^ that includes the functional gene *bla* and pUC origin (*ori*_pUC_)

The plasmid pLGC09, derived from plasmid pCR^®^4Blunt-TOPO^®^ (Invitrogen), was constructed to provide *P. pastoris* with the putative endo-β-1,3-glucanase under the control of P*cct*α (Fig. 1). The plasmid construction was done using conventional techniques, i.e. PCR, endonuclease restriction and ligation (Sambrook and Russell 2001). The restriction endonucleases used and T4 DNA ligase were purchased from New England Biolabs (Ipswich, MA, USA) and used according to the indications of the supplier. The primers used to amplify promoters, terminators, and genes are shown in Table 2. All primers were purchased from Sigma-Genosys (St. Louis, MO, USA). All PCRs were performed using *Pfu* DNA polymerase (Fermentas) in a Bio-Rad T100™ Thermal Cycler (Hercules, CA, USA). All PCRs were done under the same conditions, i.e. initial denaturation at 95 °C during 5 min and 34 cycles comprising: denaturation at 95 °C for 30 s and annealing at 55 °C for 40 s. A 2 min extension was done per expected thousand base pairs amplicon and a final extension at 72 °C for 7 min. All amplicons were verified by agarose electrophoresis and sequencing.

**Table 2:** Primers used in this study

Primer^*a*^	Sequence (5′→3′)^*b*^	Restriction enzyme	Underlined sequence^*c*^
YL1-F	GGGGtcgcgaGAATCTTCCTCAGAAGAGGAAT	*Nru*I	Homologous P*cct*α region corresponding to nucleotides (nt) 886842 to 886863
YL1-R	GCGCggatccTCACCTCCTAGGAAAAGGGTAT	*Bam*HI	Homologous P*cct*α region corresponding to nt 887263 to 887284
YL2-F	CCCCagatctCCCAAATGTCATTCTCTTCCAA	*Bgl*II	Homologous *eng* region corresponding to nt 802095 to 802116
YL2-R	GCGCctcgagTTAAACGGAATTAGCCAGTCCT	*Xho*I	Homologous *eng* region corresponding to nt 805153 to 805132
YL3-F	GCGCgtcgacTGAGTTTTAGCCTTAGACATGACT	*Sal*I	Homologous *aox1* TT region corresponding to nt 627 to 650
YL3-R	GGGGcacgtgCCGCACAAACGAAGGTCTCA	*Pml*I	Homologous *aox1* TT region corresponding to nt 970 to 957
YL4-F	GGGGatttaaatCCCACACACCATAGCTTCAAA	*Swa*I	Homologous *Ptef1* region corresponding to nt 971 to 993
YL4-R	GGGGcacgtgTCTCCAGCTTGCAAATTAAAG	*Pml*I	Homologous *cyc1* TT region corresponding to nt 2142 to 2122
YL5-F	GGGGtcgcgaGTATATTGAGGAGCAGAACTAG	*Nru*I	Homologous *leu2* region corresponding to nt 86482 to 86503
YL5-R	GGGGcacgtgCTTTTTCCGAACCTTACAGTAG	*Pml*I	Homologous *leu2* region corresponding to nt 87943 to 87922

^*a*^ *F* forward primer, *R* reverse primer

^*b*^Lowercase letters indicates the restriction endonuclease recognized site denoted in the next column

^*c*^Nucleotide numbering has been taken according to the sequence depicted in the access number mentioned in the section of materials and methods

Three modules were obtained: First, to construct the lytic module, the promoter P*cct*α amplified from the genomic DNA of *S. cerevisiae* (amplicon YL1; Table 2 shows the corresponding primers used to amplify each amplicon, i.e. YL1-F and YL1-R to amplify amplicon YL1), and the gene *eng* amplified from the genomic DNA of *P. pastoris* GS115 (amplicon YL2) were digested with* Bam*HI and* Bgl*II respectively. They were joined together through T4 DNA Ligase and PCR using primers YL1-F and YL2-R. The amplicon YL1–YL2 digested with* Xho*I and *aox1* TT amplified from pGAPZαA (Invitrogen; amplicon YL3) digested with* Sal*I were ligated together (Table 2 shows the restriction sites used to construct the module, the joining of* Bam*HI to* Bgl*II and* Xho*I to* Sal*I digested ends are cohesives but their joining does not let the sites to be regenerated), and through PCR using primers YL1-F and YL3-R the lytic module was amplified. Second, the selection marker module consisted of a fragment containing the promoters P_*tef1*_ and P_EM7_, *ble*, and the *cyc1* TT was directly amplified from pGAPZαA (amplicon YL4). Third, the full-length *leu2* gene including its promoter and transcription terminator was amplified from the genomic DNA of *P. pastoris* (amplicon YL5) to be the integration module. All modules were cloned each in pJET1.2/blunt using the CloneJET™ PCR Cloning Kit (Fermentas; Waltham, MA, USA) according to the instructions of the manufacturer. The plasmid containing the selection marker module was digested with* Pml*I, ligated to the plasmid including the integrative module digested with* Nru*I (both enzymes produce blunt ends), and amplified through PCR using the primers YL4-F and YL5-R. The two-modules amplicon was cloned in pCR^®^4Blunt-TOPO^®^ using the Zero Blunt^®^ TOPO^®^ PCR Cloning Kit for Sequencing (Invitrogen) following the instructions of the supplier. The final construction was assembled in pCR^®^4Blunt-TOPO^®^ by subcloning the fragment* Not*I–*Pml*I from the plasmid including the lytic module in* Not*I–*Swa*I digested pCR^®^4Blunt-TOPO^®^-selection marker and integration modules using classical restriction and ligation techniques (Sambrook and Russell 2001), obtaining at last the vector pLGC09 (details on the construction strategy, and pLGC09 data could be consulted in Additional files 1, 2).

### Transformation of *P. pastoris* GS115 with pLGC09

*Pichia pastoris* CL2 was obtained through transformation of electrocompetent cells of *P. pastoris* GS115 with pLGC09 as described by Higgins and Cregg (1998). A Bio-Rad MicroPulser™ (Hercules, CA, USA) was used to electroporate the cells in Sigma-Aldrich 0.2 cm gap width cuvettes (St. Louis, MO, USA). Conditions for *P. pastoris* electroporation were already programed in the apparatus (2.0 kV, 1 pulse, 4.5 ms). Ten micrograms of pLGC09 were used for 80 µl electrocompetent cells. The transformant clones were selected on YPDS agar (same as YPD, but added with 1 M sorbitol) supplemented with zeocin (500 µg/ml; Invitrogen), according to the manual included in the Invitrogen EasySelect™ *Pichia* Expression Kit.

### Effect of *eng* expression on cell morphology

The cells were observed with a microscope to determine if the *eng* expression controlled by P*cct*α and induced by cold-shock conditions had an effect on the cell wall and morphology of *Pichia* cells. The inocula of *P. pastoris* GS115 and *P. pastoris* CL2 were prepared in YPD medium [50 ml in 250 ml-Erlenmeyer flasks, 30 °C, 150 rpm, 18 h; in a TERLAB DBO incubator (El Arenal, Jal., Mexico) with orbital shaker]. The cells were harvested and washed once with sterile distilled water, centrifuged at 4000 rpm and at room temperature for 10 min in a DuPont SORVALL SUPER-ST 21 centrifuge with SL 50T rotor (Wilmington, DE, USA). The treatments described below were applied on cells grown in BMGH medium (pH 4.5; Invitrogen EasySelect™ *Pichia* Expression Kit manual) supplemented with 1/50 (v/v) of a solution containing 250 mM MnSO_4_ and 50 mM MgSO_4_. The cell pellets from centrifuged inocula were suspended in BMGH medium, adjusted to OD_600_ = 1 and 50 ml of the suspension was poured into 250 ml-Erlenmeyer flasks. The cultures were incubated at 30 °C (150 rpm) for 4 h. Cells were chilled to 4 °C for the cold-shock stage (stage I). A gene expression stage (stage II) was applied by incubating the cells at 4 °C (150 rpm) for 6 h. The cells were incubated at 30 °C (150 rpm) for 6 h (stage III) for ENG synthesis. Finally, the cells were incubated either 12 or 24 h (150 rpm) at 37 °C (stage IV) for cell lysis. Cells subjected to the entire treatment (stages I–IV) were prepared for optical microscopy as follows: cells (following stage IV incubation) were collected from 1.0 ml samples by centrifugation at 10,000 rpm for 1.5 min in a Eppendorf Centrifuge 5424 (Hamburg, Germany). After centrifugation the cells were suspended in 1.0 ml distilled water and mixed with Coomassie brilliant blue R-250 staining solution (5:1; Bio-Rad). The morphology of cells was determined with an OLYMPUS CX31 optical microscope (Shinjuku-ku, To., Japan) and the images were captured with a Sony Cyber-shot DSC-S930 digital camera (Minato-ku, To., Japan). Cell morphology was determined after 12 h in stage IV by scanning electron microscopy (SEM). Cells were sputter coated with gold and analysed by using the microscopy service of the “*Laboratorio Avanzado de Nanoscopía Electrónica”* (LANE) Cinvestav-IPN (Mexico), using a FE HRSEM-Auriga 3916 Zeiss Microscope (Oberkochen, Baden-Wurtemberg, Germany) at an accelerating voltage of 2 kV. *P. pastoris* GS115 cells served as control in the experiments and were treated in the same way as *P. pastoris* CL2 cells.

### Quantification of *eng* expression

To determine the *eng* expression, mRNA was quantified with a quantitative reverse transcription polymerase chain reaction (qRT-PCR) with an Applied Biosystems 7500 Fast Real-Time PCR System (Foster City, CA, USA). Total RNA was extracted in triplicate from culture broth samples (1 ml) of *P. pastoris* GS115 and *P. pastoris* CL2 collected at 0, 2 and 6 h during stage II; and 2 h during stage III. Cells from culture broth samples were washed with sterile distilled water as described in the previous section (except that the first and fourth samples were washed at room temperature, while the second and third samples were washed at 4 °C). The cell pellets were lyophilized and kept at −70 °C until used. Cell lysis was accomplished by three freezing cycles with liquid nitrogen and macerated with a polypropylene pestle (1 min each). Total RNA was extracted with the Trizol™ Reagent (Invitrogen) method following the instructions of the supplier. Total RNA samples were treated with DNase I (Invitrogen) to remove DNA. The first DNA strand of cDNA encoding ENG was synthesized by reverse transcription from 1 µg total RNA using the Super Script III Reverse Transcriptase System Kit (Invitrogen).

Primers and probes used for qRT-PCR analyses were designed by Applied Biosystems (Foster City, CA, USA; Table 3). The qRT-PCR analyses were done with a 7500 Fast Real-Time PCR System of Applied Biosystems using TaqMan^®^ Gene Expression Master Mix 2X (Foster City, CA, USA) with a total reaction volume of 10 μl. FAM fluorophore was used to detect both *act1* (housekeeping gene control) and *eng.* Amplification conditions for qRT-PCR were as follows: 50 °C for 2 min, 95 °C for 10 min, 40 cycles of 95 °C for 15 s followed by 60 °C for 1 min. All reactions were performed in triplicate on each plate with three independent replicates, and gene expression values were calculated using the difference in *eng* expression relative to *act1* expression using the method 2^−ΔΔCt^ (Livak and Schmittgen 2001).

**Table 3:** Real-time PCR primers and probes used in this study

Primer or probe^*a*^	Sequence (5′–3′)	Modification^*b*^
Primer		
*eng*-F (TaqMan MGB)	GTCGGTTGACGGGTTAATTGTG	None
*eng*-R (TaqMan MGB)	AGCCGCATAGTCGTAGTAAATCA	None
*act1*-F (TaqMan MGB)	TGTCTGAGCGTGGTTACACTTTT	None
*act1*-R (TaqMan MGB)	CAACGTAACAAAGCTTCTCCTTGAT	None
Probe		
*eng* (TaqMan® probe)	CTGCCTTGGCAACTTG	FAM/MGB-NFQ
*act1* (TaqMan® probe)	CACGGACGATTTCTCT	FAM/MGB-NFQ

*FAM* fluorophore 6-carboxyfluorescein, *MGB* minor groove binder, *NFQ* nonfluorescent quencher

^*a*^ *F* forward primer, *R* reverse primer, *eng* gene encoding a putative endo-β-1,3-glucanase, *act1* gene encoding actin 1

^*b*^Reporter 1 dye/reporter 1 quencher

## Results

### Effect of *eng* expression on cell morphology

**Fig. 2:**
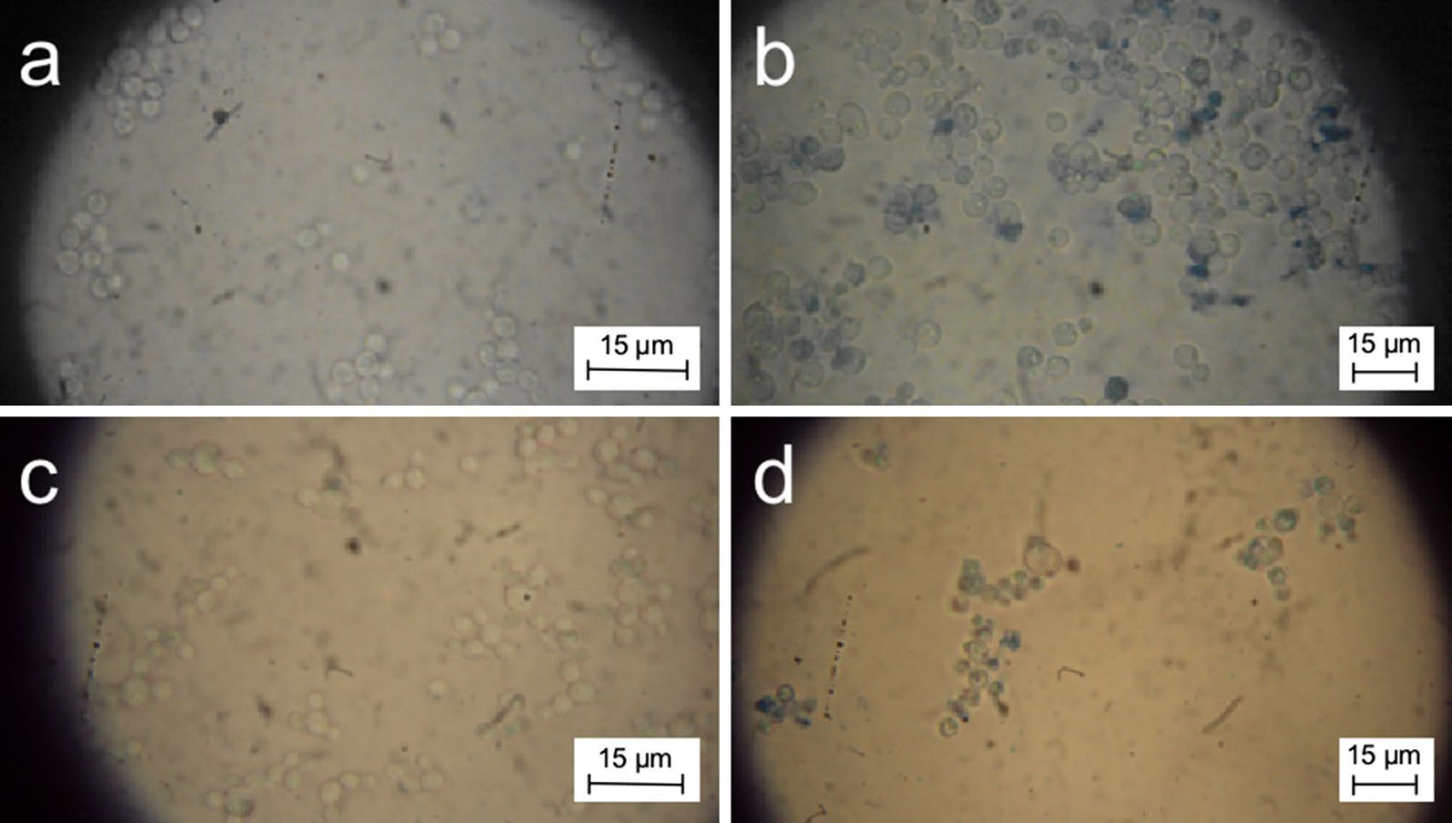
Analysis by optical microscopy (100×) after staining with Coomassie* blue* R-250 of *Pichia pastoris* GS115 (control) and *P. pastoris* CL2 cells induced by cold-shock and after the entire treatment. **a**, **c** Control cells of *P. pastoris* GS115 at 12 h and 24 h in stage IV, respectivelly; **b**, **d** *P. pastoris* CL2 cells at 12 and 24 h in stage IV, respectively. *Bar* 15 µm

After the transformation of *P. pastoris* GS115 with pLGC09, transgenic *P. pastoris* CL2 was selected among six zeocin resistant colonies. In addition, *P. pastoris* CL2 strain was the most resistant to the antibiotic, the most sensitive to cold-shock induction and showed the most aberrant cell morphology as determined by optical microscopy with Coomasie brilliant blue staining. Figure 2 shows *P. pastoris* CL2 cells, 12 and 24 h after a treatment consisting of the cold-shock (stage I), gene expression (stage II), ENG synthesis (stage III) and lysis (stage IV). *Pichia pastoris* GS115 was used as control. The cells of *P. pastoris* CL2 increased in size and became irregular after 12 h in stage IV. Some cells were stained with Coomasie brilliant blue, and cellular debris were also stained with the dye (Fig. 2b). Twenty-four hours after the treatment, most cells were lysed and those that were not broken were irregular and enlarged. The latter were stained and cellular debris was noticed clearly (Fig. 2d). Contrarily, the *P. pastoris* GS115 cells were not affected noticeably by the treatment (Fig. 2a, c). The morphology of each cell type was also examined before applying the entire treatment, but no differences were found in morphology or fragility, between the *P. pastoris* GS115 cells and *P. pastoris* CL2 cells (data not shown).

**Fig. 3:**
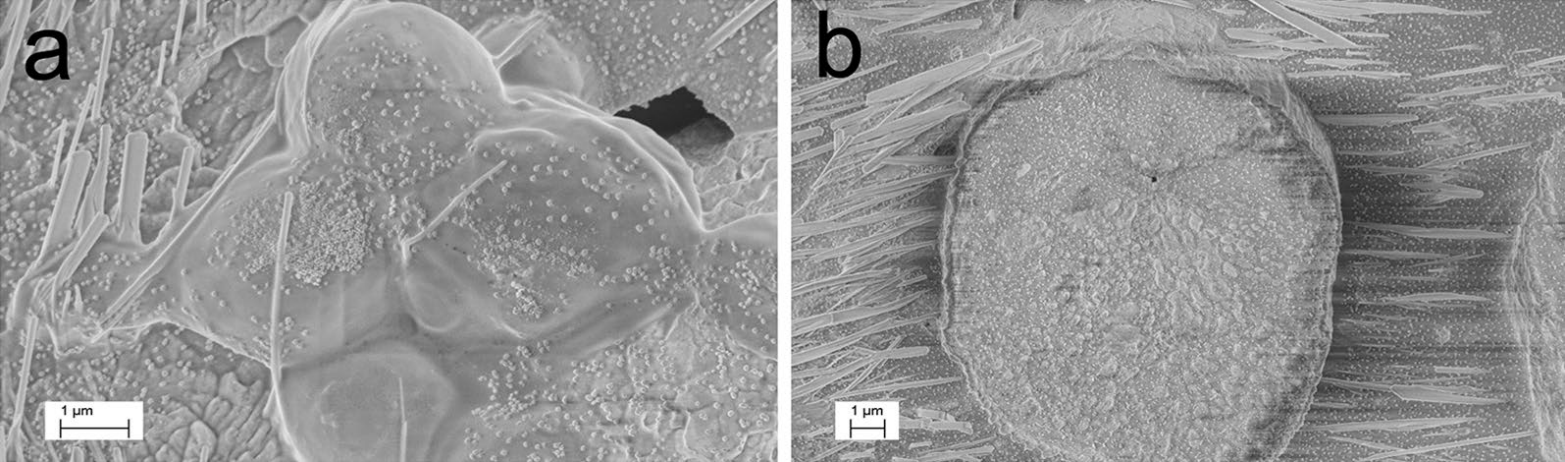
Analysis by scanning electron microscopy (SEM) of *Pichia pastoris* GS115 (control) and *P. pastoris* CL2 cells induced by cold-shock and after the entire treatment. **a** Control cells of *P. pastoris* GS115 at 12 h in stage IV. **b** *P. pastoris* CL2 at 12 h during stage IV. In both photographs phosphate salt crystals are observed surrounding cells. *Bar* 1 µm

Additionally, *Pichia pastoris* GS115 cells and transformed cells of *P. pastoris* CL2, both subjected to the entire treatment were prepared (12 h incubation on stage IV) and observed by SEM. The *Pichia pastoris* GS115 control cells had an average size ranging from 2 to 3 μm (Fig. 3a), whereas *P. pastoris* CL2 cells formed spheroplasts larger than 10 μm (Fig. 3b).

### Quantification of *eng* expression

**Fig. 4:**
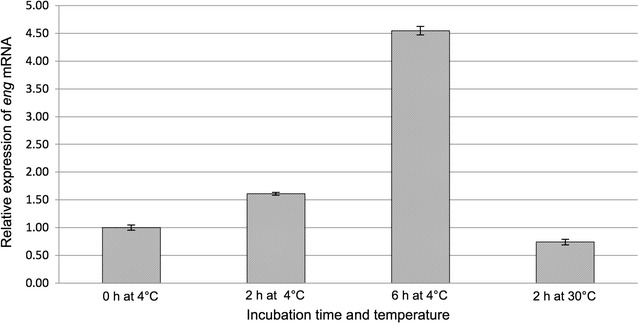
Relative expression of *eng* mRNA in *P. pastoris* CL2 in response to cold-shock at 4 °C. CL2 cells were grown in medium BMGH (30 °C for 4 h), from OD_600_ = 1 inoculum, then subjected to cold-shock for the induction of *eng* mRNA transcript expression (4 °C for 6 h). Samples were taken at 0, 2 and 6 h for analysis by qRT-PCR; and finally, they were transferred to 30 °C for 4 h for ENG product synthesis, stage in which the corresponding sample at 2 h was taken for analysis by qRT-PCR. The values of relative expression of *eng* mRNA were calculated in relation to *eng* and the housekeeping constitutive gene *act1* (control) expression using method 2^−ΔΔCt^

qRT-PCR analyses were done to determine the expression of *eng* induced in *P. pastoris* CL2. Figure 4 shows the relative expression of *eng* in *P. pastoris* CL2 at different stages of treatment as compared with *eng* expression in *P. pastoris* GS115 under the same conditions. The relative expression of the *eng* within stage II was 1.6 fold higher at 4 °C after 2 h and 4.6 fold after 6 h. During the stage III (at 30 °C for 2 h), the relative expression of *eng* was lower, i.e. 0.74 times.

## Discussion

The biotechnological and pharmaceutical industries are fast-growing (Rader 2013; Tang and Zhao 2009). In particular, substantial resources are applied to the development of recombinant protein production with *P. pastoris* (Calik et al. 2015), the most widely used system as expression system for heterologous protein production (Gasser et al. 2013). Purification of recombinant proteins, including cell lysis is commonly recognized among the most costly parts of entire production processes (Ernst et al. 1997). Additionally, the cells are disrupted frequently under harsh conditions that can affect the product quality. A low cost process for cell lysis that does not compromise protein quality or the environment would be desirable. Autolysis of *P. pastoris* induced at low temperature could be such a technique.

The recovery of an intracellular recombinant protein depends on breaking the cell wall at the end of yeast growth. The inner layer of the cell wall of *S. cerevisiae* is formed by glucanes with β-1,3 and β-1,6 glycosidic bonds and chitin (Levin 2011). In *Pichia* the glucanes: chitin ratio varies from 75:25 to 95:5 depending on growth conditions (Chagas et al. 2014; Farinha et al. 2015). Glucanes form a net that is responsible for the rigidity and shape of yeast cells (Levin 2011). An enzyme degrading glucanes would weaken the yeast cell wall and compromise the cell integrity. Tanaka and Phaff (1965) found that β-glucanases from *Bacillus circulans* are able to degrade baker’s yeast cell wall and that β-1,3-glucanase was more effective than the β-1,6-glucanase. Moreover, yeasts possess glucanase encoding genes within their genomes, for these enzymes are needed to restructure the cell wall and to separate the budding cells from the mother cell (Baladrón et al. 2002; Martín-Cuadrado et al. 2003). *P. pastoris* has an *eng* gene encoding a putative endo-β-1,3-glucanase homologous to *eng1* from *S. cerevisiae* (Baladrón et al. 2002). In the present work, this endogenous *eng* gene was selected to show that its overexpression in *P. pastoris* induces cell wall degradation, causing cell fragility as well as cell lysis.

Somer et al. (2002) found that the expression of P*cct*α of *S. cerevisiae* cells under cold-shock conditions increased three to four times. In *P. pastoris* CL2, the expression of *eng* was 3.6 times higher under cold-shock conditions than in absence of the induction process. This proved that P*cct*α was functional in *P. pastoris* CL2 as it is in its natural genetic environment (*S. cerevisiae*). The return to warmer conditions after the cold-shock decreased rapidly the amount of the RNA transcripted under P*cct*α control suggesting a rapid degradation of *eng* mRNA in this system. Somer et al. (2002) obtained similar results in their study.

To our knowledge, cold-shock promoters are not used yet in yeasts intended for industrial use. However, these promoters are already used in prokaryotes, especially in *E. coli*. The most often used cold-shock vector includes the promoter of the *E. coli* endogenous gene *cspA*, which is suitable for induction of cloned genes by means of cold-shock. The promoter is used mainly to express proteins that are toxic to the host, or for highly unstable systems at 37 °C (Mujacic et al. 1999) to confer higher solubility and stability to expressed proteins (Qing et al. 2004).

After the entire treatment transgenic *P. pastoris* CL2 cells were irregular and ranged in size from similar to 3.3 times larger than the control cells. This appears to be due to the loss of the glucanes-net integrity resulting in spheroplasts. Coomasie blue staining showed that *P. pastoris* CL2 cells became permeable to the dye, but staining was absent in the control cells. This also indicated a loss of cell wall integrity. In *P. pastoris* CL2, the amount of cell debris increased after the entire treatment compared to the untreated control cells suggesting that the treated cells became fragile and were easily broken up. In fact, the treated cells could not be washed, as they had become too fragile and were broken up. Scan-electronic microscopy confirmed that spheroplasts of *P. pastoris* CL2 cells were bigger (~10 µm diameter) than the control cells (~3 µm diameter).

In this work it was shown that a transgenic strain of *P. pastoris* was constructed, which is able to undergo autolysis upon induction by cold-shock. The P*cct*α cold-shock promoter from *S. cerevisiae* is functional in the genetic expression system of *P. pastoris*. The overexpression of the endogenous *eng* gene of *P. pastoris* leads to cellular fragility and autolysis. This work proves that the *P. pastoris eng* gene integrated to a suitable yeast expression system including the *S. cerevisiae* P*cct*α can be used to develop an innovative inducible autolysis system by cold-shock. This approach might be compatible with clean technologies, and with further development of particular applications might be a way to improve production processes for recombinant proteins, enzymes and pharmaceutical products avoiding mechanical or chemical cell lysis. Other stronger cold-shock promoters and enzymes with higher lytic activity could be studied to increase the effectiveness of the autolytic system.

## Additional files

**Additional file 1.** Construction strategy of pLGC09.

**Additional file 2.** Plasmid pLGC09.

## Abbreviations

ENG: putative endo-β-1,3-glucanase
*eng*: gene encoding a putative endo-β-1,3-glucanase
*cct*α: gene encoding alpha subunit from cytoplasmic chaperonin CCT
CCTα: alpha subunit from CCT
P*cct*α: promoter of the alpha subunit of CCT
*ble*: zeocin resistance gene
ori_pUC_: pUC replication origin
*leu2*: functional gene encoding β-isopropylmalate dehydrogenase
P_*tef1*_: translational elongation factor 1 gene promoter
P_EM7_: synthetic prokaryotic promoter
*aox1* TT: *aox1* transcription termination region
*cyc1* TT: *cyc*1 transcription termination region
