# Correction to: Autolysis of *Pichia pastoris* induced by cold

**DOI:** 10.1186/s13568-018-0712-2

**Published:** 2018-11-14

**Authors:** Yaneth Bartolo-Aguilar, Luc Dendooven, Cipriano Chávez-Cabrera, Luis B. Flores-Cotera, María E. Hidalgo-Lara, Lourdes Villa-Tanaca, Rodolfo Marsch

**Affiliations:** 1 Department of Biotechnology and Bioengineering, Cinvestav-IPN, Av. Instituto Politécnico Nacional 2508, Col. San Pedro Zacatenco, 07360 Gustavo A. Madero, CDMX Mexico; 2 Department of Microbiology, Escuela Nacional de Ciencias Biológicas del IPN, Prol. Carpio y Plan de Ayala S/N Col. Santo Tomás, 11340 Miguel Hidalgo, CDMX Mexico

## Correction to: AMB Expr (2017) 7:95 10.1186/s13568-017-0397-y

The original version of this article (Bartolo-Aguilar et al. 2017) was written and published including the first construction strategy of pLGC09, but not the final one. This error was pointed out by a reader and an analysis of sequences of parts of the plasmid corroborated this. The final construction strategy was reanalysed and confirmed the error. This error affected the text, Table 2, Fig. 1 and the Additional files, but did not affect the results and conclusions stated in the paper. The authors regret that this error occurred in the original publication of the article. The corrected text, Table 2 and Fig. 1, and the Additional files (Additional file 1. Construction strategy of pLGC09 and Additional file 2. Plasmid pLGC09) are given in this correction.

**Table 2:** Primers used in this study

Primer^a^	Sequence (5′→3′)^b^	Restriction enzyme	Underlined sequence^c^
YL1-F	GGGGtcgcgaAATCTTCCTCAGAAGAGGAAT	*Nru*I	Homologous P*cctα* region corresponding to nucleotides (nt) 886843 to 886863
YL1-R	GCGCggatccTGAATAATTGGGACATCGCTAA	*Bam*HI	Homologous P*cctα* region corresponding to nt 887226 to 887247
YL2-F	CCCCagatctTTCTCTCGTTAGCTTCCCAAA	*Bgl*II	Homologous *eng* region corresponding to nt 802080 to 802100
YL2-R	GCGCctcgagGTCAACTATGTGTGGTACCTT	*Xho*I	Homologous *eng* region corresponding to nt 805196 to 805216
YL3-F	GCGCgtcgacTGAGTTTTAGCCTTAGACATGACT	*Sal*I	Homologous *aox1* TT region corresponding to nt 890 to 913
YL3-R	GGGGatttaaatGGGGATCCGCACAAACGAA	*Swa*I	Homologous *aox1* TT region corresponding to nt 1217 to 1235
YL4-F	CCCCatttaaatGGATCCCCCACACACCATA	*Swa*I	Homologous *Ptef1* region corresponding to nt 965 to 983
YL4-R	GCGCcacgtgCACATGTTGGTCTCCAGCTT	*Pml*I	Homologous *cyc1* region corresponding to nt 2157 to 2138
YL5-F	GGGGgccggcCCAAGTCCAAGGTCTCCAAT	*Nae*I	Homologous *leu2* region corresponding to nt 86424 to 86443
YL5-R	GGGGcacgtgTTTCCGAACCTTACAGTAGAG	*Pml*I	Homologous *leu2* region corresponding to nt 87940 to 87920

^a^*F* forward primer, *R* reverse primer

^b^Lowercase letters indicates the restriction endonuclease recognized site denoted in the next column

^c^Nucleotide numbering has been taken according to the sequence depicted in the access number mentioned in the section of Materials and methods

**Fig. 1:**
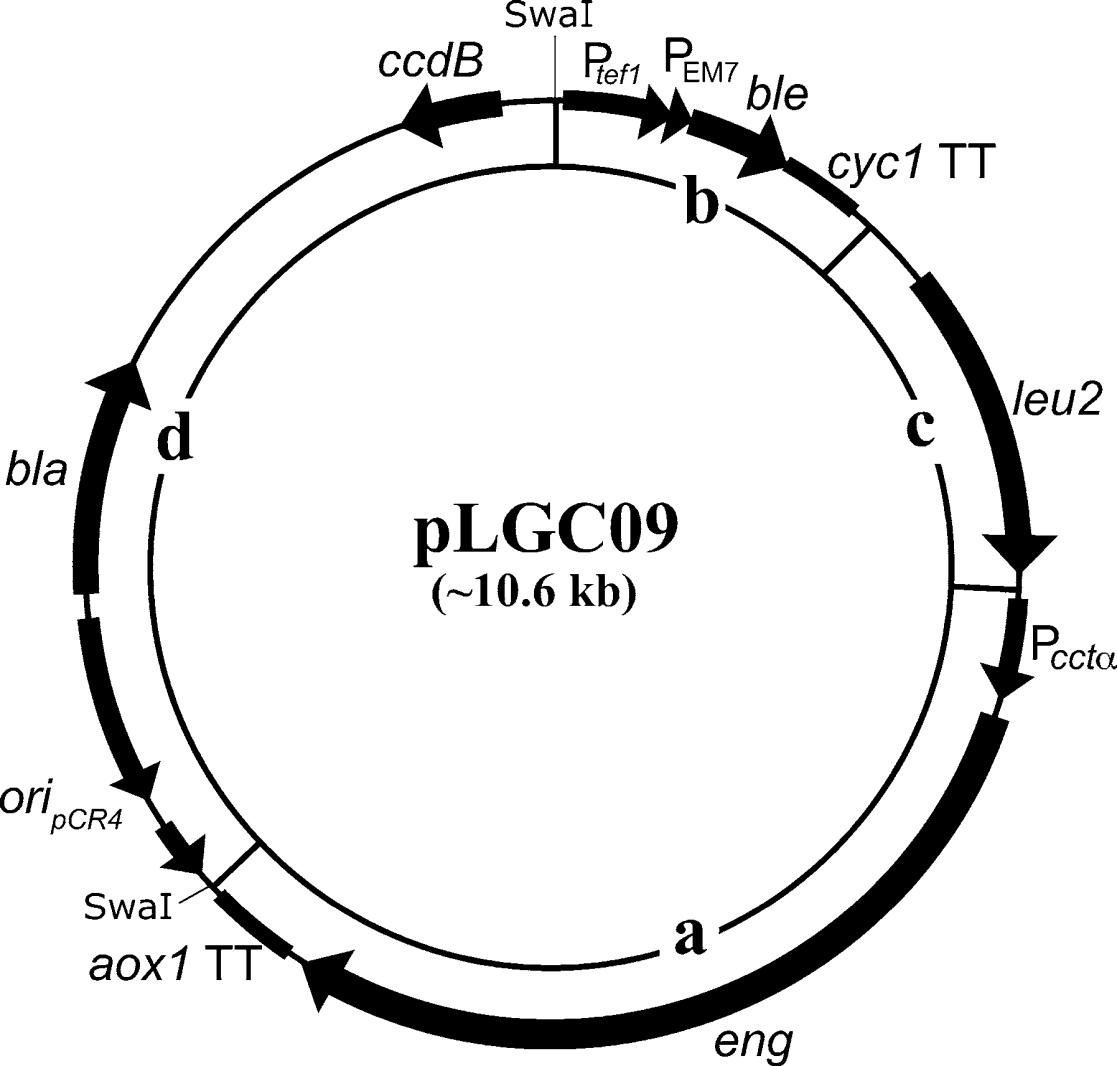
Structure of pLGC09. Plasmid pLGC09 comprises three modules: **a** the lytic regulated module is composed of the promoter of chaperonin CCT one complex (P*cct*α) that drives expression of *eng* in *P. pastoris* and it is finished by *aox1* transcription termination (*aox1* TT) region; **b** the selection marker module is composed of the translational elongation factor 1 gene promoter (P_*tef1*_) and of the EM7 synthetic prokaryotic promoter (P_EM7_) that drive expression of the *ble* gene in *P. pastoris* and *E. coli* respectively, these expressions are finished by *cyc1* transcription termination region (*cyc1* TT); **c** the homologous recombination module is composed of the *leu2* functional gene including its promoter and transcription termination region; and **d** the replicon pCR^®^4Blunt-TOPO^®^ that includes the functional gene *bla* and pUC origin (*ori*_pUC_)

The second paragraph in the section Materials and methods entitled, “Construction of plasmid pLGC09” was wrong and should read:

“Three modules were obtained: First, to construct the lytic module, the promoter P*cctα* amplified from the genomic DNA of *S. cerevisiae* (amplicon YL1; Table 2 shows the corresponding primers used to amplify each amplicon, i.e. YL1-F and YL1-R to amplify amplicon YL1) was cloned in pCR^®^4Blunt-TOPO^®^ (using the Zero Blunt^®^ TOPO^®^ PCR Cloning Kit for Sequencing (Invitrogen) following the instructions of the supplier; pLGC01), the gene *eng* amplified from the genomic DNA of *P. pastoris* GS115 (amplicon YL2) was cloned in *Pme*I digested pLGC01 using T4 DNA Ligase to obtain pLGC03. This plasmid digested with *Xho*I and *aox*1 TT amplified from pGAPZαA (Invitrogen; amplicon YL3) digested with *Sal*I were ligated together (Table 2 shows the restriction sites used to construct the module, the joining of *Xho*I to *Sal*I digested ends are cohesive but their joining does not let the restriction sites to be regenerated), and amplified using primers YL1-F and YL3-R (amplicon YL1-YL3; lytic module). The amplicon was cloned in pCR^®^4Blunt-TOPO^®^ (pLGC05). Second, the selection marker module consisted of a fragment containing the promoters P_*tef*1_ and P_EM7_, *ble*, and the *cyc*1 TT was directly amplified from pGAPZαA (amplicon YL4). Third, the full-length *leu2* gene including its promoter and transcription terminator was amplified from the genomic DNA of *P. pastoris* (amplicon YL5) to be the integration module. The last two modules were cloned each in pJET1.2/blunt using the Clone-JET™ PCR Cloning Kit (Fermentas; Waltham, MA, USA; pLGC06 and pLGC07, respectively) according to the instructions of the manufacturer. The plasmid containing the selection marker module was digested with *Pml*I, ligated to the plasmid including the integrative module digested with *Nae*I (both enzymes produce blunt ends), and amplified through PCR using the primers YL4-F and YL5-R. The two-modules amplicon was cloned in pCR^®^4Blunt-TOPO^®^ (pLGC08). The final construction was assembled by subcloning the fragment *Not*I–*Pml*I from the plasmid including the selection marker and integration modules in *Not*I–*Nru*I digested pCR^®^4Blunt-TOPO^®^-lytic module using classical restriction and ligation techniques (Sambrook and Russell 2001), obtaining at last the vector pLGC09 (details on the construction strategy, and pLGC09 data could be consulted in Additional files 1, 2).”

Table 2 was corrected, including the sequences of the primers used to construct pLGC09.

These corrections do not alter any conclusions or results of our study.

The authors wish to apologize for this confusion and the inconvenience that this might have caused.

## Additional files

**Additional file 1.** Construction strategy of pLGC09.

**Additional file 2.** Plasmid pLGC09.
